# Conversion chemoradiotherapy combined with nab-paclitaxel plus cisplatin in patients with locally advanced borderline-resectable or unresectable esophageal squamous cell carcinoma: a phase i/ii prospective cohort study

**DOI:** 10.1007/s00066-024-02286-8

**Published:** 2024-08-12

**Authors:** Nuo Yu, Xiankai Chen, Jiao Li, Xiaozheng Kang, Zhen Wang, Ruixiang Zhang, Jianjun Qin, Yong Li, Qingfeng Zheng, Guojie Feng, Lei Deng, Tao Zhang, Wenqing Wang, Wenyang Liu, Jianyang Wang, Qinfu Feng, Jima Lv, Zongmei Zhou, Zefen Xiao, Nan Bi, Yin Li, Xin Wang

**Affiliations:** 1 Department of Radiation Oncology, National Cancer Center/National Clinical Research Center for Cancer/Cancer Hospital, Chinese Academy of Medical Sciences and Peking Union Medical College, No.17 Panjiayuan Nanli, Chaoyang District, 100021 Beijing, China; 2 Department of Thoracic Surgery, National Cancer Center/National Clinical Research Center for Cancer/Cancer Hospital, Chinese Academy of Medical Sciences and Peking Union Medical College, No.17 PanjiayuanNanli, Chaoyang District, 100021 Beijing, China

**Keywords:** Nab-paclitaxel, Esophageal squamous cell carcinoma, Conversion chemoradiotherapy, Esophagectomy, Postoperative complications

## Abstract

**Background:**

To evaluate the efficacy and safety of nab-paclitaxel plus cisplatin as the regimen of conversional chemoradiotherapy (cCRT) in locally advanced borderline resectable or unresectable esophageal squamous cell carcinoma (ESCC).

**Methods:**

Patients with locally advanced ESCC (cT3‑4, Nany, M0‑1, M1 was limited to lymph node metastasis in the supraclavicular area) were enrolled. All the patients received the cCRT of nab-paclitaxel plus cisplatin. After the cCRT, those resectable patients received esophagectomy; those unresectable patients continued to receive the definitive chemoradiotherapy (dCRT). The locoregional control (LRC), overall survival (OS), event-free survival (EFS), distant metastasis free survival (DMFS), pathological complete response (pCR), R0 resection rate, adverse events (AEs) and postoperative complications were calculated.

**Results:**

45 patients with ESCC treated from October 2019 to May 2021 were finally included. The median follow-up time was 30.3 months. The LRC, OS, EFS, DMFS at 1 and 2 years were 81.5%, 86.6%, 64.3%, 73.2 and 72.4%, 68.8%, 44.8%, 52.7% respectively. 21 patients (46.7%) received conversional chemoradiotherapy plus surgery (cCRT+S). The pCR rate and R0 resection rate were 47.6 and 84.0%. The LRC rate at 1 and 2 years were 95.0%, 87.1% in cCRT+S patitents and 69.3%, 58.7% in dCRT patients respectively (HR, 5.14; 95%CI, 1.10–23.94; *P* = 0.021). The toxicities during chemoradiotherapy were tolerated, and the most common grade 3–4 toxicitiy was radiation esophagitis (15.6%). The most common postoperative complication was pleural effusion (38.1%) and no grade ≥ IIIb complications were observed.

**Conclusion:**

nab-paclitaxel plus cisplatin are safe as the regimen of conversional chemoradiotherapy of ESCC.

**Supplementary Information:**

The online version of this article (10.1007/s00066-024-02286-8) contains supplementary material, which is available to authorized users.

## Background

Esophageal cancer is a malignant tumor with high morbidity and mortality, and China accounted for the highest burden [1]. In China, esophageal squamous cell cancer (ESCC) is the most common histological type, accounting for > 90% of the total [2].

For patients with unresectable stage-T4b ESCC, the National Comprehensive Cancer Network (NCCN) and Chinese Society of Clinical Oncology (CSCO) recommend definitive chemoradiotherapy (dCRT). Consensus on neoadjuvant therapy for patients with borderline-resectable ESCC is lacking. Some studies have explored the role of neoadjuvant chemotherapy or cisplatin and 5‑fluorouracil (PF)-based neoadjuvant chemoradiotherapy (nCRT) in this subset of patients [3, 4]. Some studies have demonstrated that, if patients with unresectable ESCC can receive R0 resection after conversion therapy, then they would have a significantly better prognosis than patients who have unresectable ESCC [3–6].

For dCRT and nCRT, paclitaxel plus platinum is the preferred combination used concurrently with radiotherapy. Nanoparticle albumin-bound paclitaxel (nab-paclitaxel) is a brand-new preparation. nab-paclitaxel can bind to tumor cells *via* secreted protein acidic and rich in cysteine (SPARC) and enter tumor cells, releasing drugs that kill tumor cells [7]. Compared with traditional solvent-based paclitaxel (sb-paclitaxel), pretreatment with nab-paclitaxel is not required and few allergic reactions occur. nab-paclitaxel has been recommended by several guidelines for the treatment of advanced tumors such as cancer of the breast, lung, and pancreas. nab-paclitaxel has been used widely in radiotherapy-, chemotherapy- and immunotherapy-related studies of ESCC in China [8–10]. However, studies on the use of nab-paclitaxel in conversion chemoradiotherapy (cCRT) and its effect on postoperative outcomes in ESCC are lacking.

This phase I/II study aimed to evaluate the efficacy and safety of nab-paclitaxel plus cisplatin as a regimen of cCRT in patients with borderline-resectable or unresectable ESCC.

## Materials and methods

### Inclusion criteria

The inclusion criteria were patients: (1) aged 18–75 years; (2) with thoracic ESCC proven by pathology or cytology; (3) with clinical stages T3N + M0‑1 and T4N0 / + M0‑1 (according to American Joint Committee on Cancer, version 8) with M1 limited to metastasis to the supraclavicular lymph nodes; (4) with Eastern Cooperative Oncology Group performance status score 0–1; (5) for whom no other anticancer therapy was carried out before enrollment; (6) with normal hematologic, hepatic, and renal functions.

### Evaluation of clinical staging

**Fig. 1:**
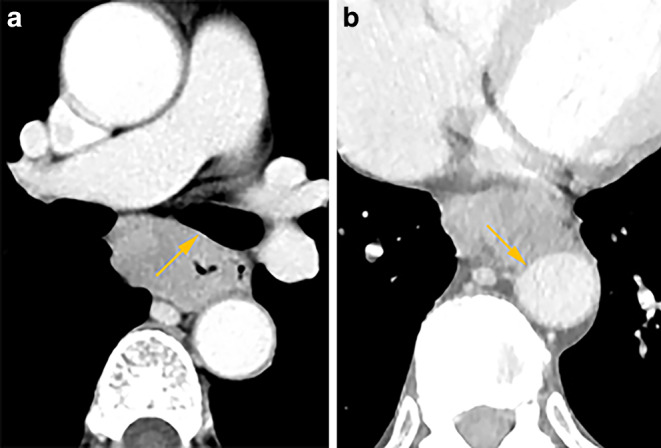
Typical CT images of borderline-resectable (**a**) and unresectable tumors (**b**). **a** shows the fatty plane between the primary tumor and left main bronchus to be blurred. **b** shows that the aorta is encased rigidly by a tumor with an interface arc between the tumor and aortic wall ≥ 90°

The clinical stage was determined by imaging: cervical and thoracoabdominal enhanced computed tomography (CT), endoscopy, endoscopic ultrasonography (EUS), bronchoscopy, positron emission tomography-computed tomography (PET-CT), and/or magnetic resonance imaging (MRI). The diagnostic criteria of borderline-resectable tumors were: (i) EUS indicated that the primary tumor had broken through the adventitia, and the interface between the primary tumor and tracheobronchial tree (TT) or aorta was unclear; (ii) CT or MRI showed that the fatty plane between the tumor (primary tumor or metastatic lymph nodes) and TT or aorta was blurred; (iii) TT deformity due to compression by the tumor but not diagnosed as organ invasion. Invasion of the TT, aorta, or recurrent laryngeal nerve leading to hoarseness was considered to denote unresectable disease. TT invasion was detected by biopsy findings from bronchoscopy, or tumor (primary tumor or metastatic lymph nodes) protruding significantly into the TT as confirmed by CT. Aortic invasion was assessed by CT and/or MRI showing that the aorta was encased rigidly by the tumor with an interface arc between the tumor and aortic wall ≥ 90° ([11]; Fig. 1). shows some typical CT images of borderline-resectable and unresectable tumors from our study.

### CRT

Radiotherapy consisted of the planning gross tumor volume (PGTV) and planning target volume (PTV) being administered at 44.94 and 37.8 Gy, in 21 daily fractions of 2.14 and 1.8 Gy, respectively. If the lesion remained unresectable after cCRT, then dCRT was undertaken to a total dose of 59.92/50.4 Gy in 28 fractions. Simultaneous integrated boost radiotherapy (SIB-RT) was undertaken during intensity-modulated radiation therapy/volumetric-modulated arc therapy with a 6-MV photon beam. Concurrent chemotherapy involved infusion of nab-paclitaxel (100 mg per time) and cisplatin (25 mg/m^2^) on days 1, 8, 15, 22, and 29. Adverse events were evaluated by the National Cancer Institute Common Terminology Criteria for Adverse Events, version 5.0.

### Surgery

If curative resection was considered by surgeons based on enhanced CT once cCRT had been completed, the patient was scheduled for surgery. Minimally invasive or open McKeown esophagectomy combined with a two- or three-field lymphadenectomy (only if metastatic supraclavicular lymph nodes presented) was undertaken 6–8 weeks after completion of cCRT. A gastric tube was reconstructed using a posterior-mediastinum route. Postoperative complications were described according to the Clavien–Dindo classification.

### Endpoints and statistical analyses

The goal of the phase I trial was to estimate the dose-limiting toxicity (DLT) of nab-paclitaxel with cisplatin concurrently with radiotherapy. Concurrent chemotherapy was terminated without dose modification if DLT occurred.

DLT was defined as any of the following: (i) leukopenia or neutropenia of grade ≥ 3; (ii) thrombocytopenia or anemia of grade ≥ 3; (iii) non-hematologic toxicity of grade ≥ 3. The study was stopped if the number of DLTs was ≥ 3 out of 3, 3 out of 4, 4 out of 5 or 6, 5 out of 7 or 8, or 6 out of 9 or 10 patients. This was a Pocock-type stopping boundary that yielded the greatest probability of crossing the boundary (5%) when the prevalence of DLT was acceptable (20%).

The aim of the phase II trial was to determine the safety and efficacy of this regimen. The primary endpoint was 1‑year locoregional control (LRC), defined as relapse of the tumor (primary, tumor bed, anastomosis, and/or regional lymph nodes). The 1‑year prevalence of LRC was predicted to be improved from 70 to 81% based on a SIB-RT study in ESCC, with one-sided α = 0.05 and 80% power [12]. Ninety-six patients were required. Assuming that 10% of patients dropped out, then 107 patients were needed for the phase II study.

Secondary endpoints were the prevalence of R0 resection, pathologic complete response (pCR), overall survival (OS; time from the start of cCRT to death from any cause), event-free survival (EFS; time from radiotherapy initiation to any event, including locoregional recurrence, distant metastasis, or death from any cause), distant metastasis–free survival (DMFS; time from cCRT initiation to the first distant metastasis, including non-regional lymph nodes and organs, or death from any cause).

Statistical analyses were done using R (R Foundation for Statistical Computing, Vienna, Austria) and SPSS 26.0 (IBM, Armonk, NY, USA). LRC, OS, EFS, and DMFS were calculated by the Kaplan–Meier method and compared using the log-rank test. *P* < 0.05 was considered significant.

## Results

### Characteristics of patients

**Fig. 2:**
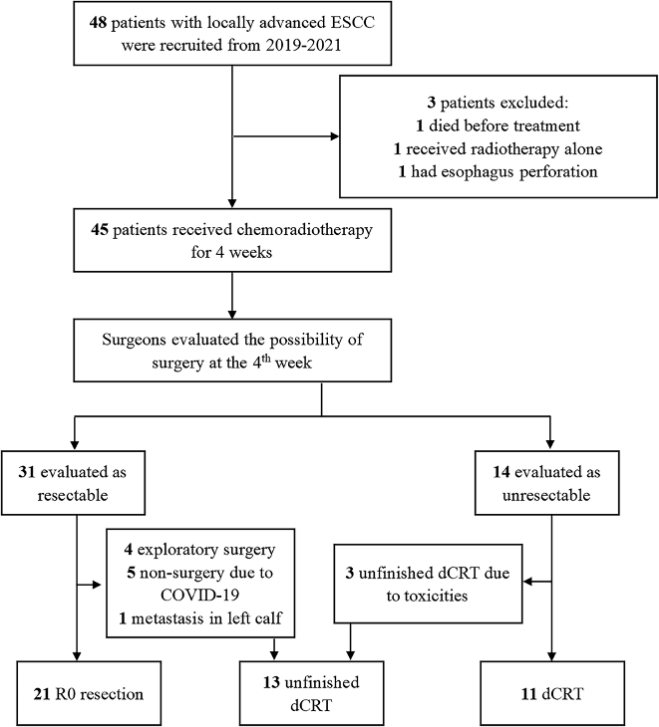
CONSORT diagram. Abbreviations: *ESCC* esophageal squamous cell carcinoma, *cCRT* conversion chemoradiotherapy, *dCRT* definitive chemoradiotherapy

Between March 2020 and May 2021, 48 patients were screened for this clinical trial from which 45 patients (10 in phase I and 35 in phase II) were enrolled in the study. The phase II study was closed because of slow accrual caused by the coronavirus disease-2019 (COVID-19) pandemic. Figure 2 shows the CONSORT diagram. The characteristics of patients at baseline are shown in Table 1. There were 39 males and six females with a median age of 60 (range, 47–70) years. Clinical stages III and IV were reported in 51.1 and 48.9% of patients, respectively.

**Table 1:** Baseline Characteristics

Characteristics	No. of paitents (%)
All	45 (100.0)
*Gender*	
Male	39 (86.7)
Female	6 (13.3)
*Age (years)*	
Mean ± SD	60.24 ± 5.47
Range	47–70
*ECOG PS*	
0	4 (8.9)
1	41 (91.1)
*Tumor length (cm*^*3*^*)*	
≤ 5	25 (55.6)
> 5	20 (44.4)
*Smoking*	
Yes	33 (73.3)
No	12 (26.7)
*Drinking*	
Yes	31 (68.9)
No	14 (31.1)
*Tumor location*	
Upper thoracic	7 (15.6)
Middle thoracic	18 (40.0)
Lower thoracic	20 (44.4)
*T stage*	
T3	30 (66.7)
T4a	8 (17.8)
T4b	7 (15.6)
*N stage*	
N0	1 (2.2)
N1	16 (35.6)
N2	21 (46.7)
N3	7 (15.6)
*M stage*	
M0	37 (82.2)
M1	8 (17.8)
*cTNM stage*	
III	23 (51.1)
IVA	14 (31.1)
IVB	8 (17.8)
*GTV Volume, cm*^*3*^	
Mean ± SD	48.3 ± 21.9
Range	12.2–100.7
*PTV Volume, cm*^*3*^	
Mean ± SD	483.8 ± 118.1
Range	273.9–730.3
*Cycles of Chemotherapy*	
1–2	12 (26.7)
3–5	33 (77.3)

*ECOG PS* Eastern Cooperative Oncology Group performance status, *GTV* gross tumor volume, *PTV* planning target volume

### Toxicity and treatment compliance

#### Phase I

The phase I portion of our clinical trial comprised 10 patients. The toxicities observed in these patients are summarized in Supplemental Table S1. Three of 10 patients developed DLTs. The safety of this regimen was confirmed in phase I and was adopted in the phase II study.

#### Phase II

An additional 35 patients were enrolled in the phase II portion, and 45 patients were evaluated. The toxicities experienced by 45 patients during CRT are stated in Table 2. Of 45 patients, 44 patients (97.8%) experienced any grade of toxicities, and 11 patients (24.4%) suffered toxicities of grade 3–4. The most common grade 3–4 toxicity was radiation esophagitis, which was observed in seven patients (15.6%).

**Table 2:** Adverse events during chemoradiotherapy

Adverse events	Any Grade (%)	Grade 3–4 (%)
*Any adverse event*	44 (97.8)	11 (24.4)
*Nonhematological toxicity*		
Fatigue	5 (11.1)	0
Nausea	8 (17.7)	0
Anorexia	6 (13.3)	0
Diarrhea	3 (6.7)	0
Cough	3 (6.7)	0
Radiation esophagitis	44 (97.8)	7 (15.6)
Radiation pneumonitis	0	0
Radiation dermatitis	12 (26.7)	0
*Hematological toxicity*		
Anemia	29 (64.4)	0
Neutropenia	24 (53.3)	2 (4.4)
Thrombocytopenia	0	0
Leukopenia	41 (91.1)	4 (8.9)
Increase in ALT/AST	5 (11.1)	1 (2.2)
Hypoalbuminemia	29 (64.4)	0

Thirty-three patients (73.3%) completed 3–5 cycles of concurrent chemotherapy. Twelve patients completed 1–2 cycles of chemotherapy because of grade 3 radiation esophagitis (four, 8.9%), grade 3 leukopenia (four, 8.9%), grade 1 fever (three, 6.7%) or grade 3 fever (one, 2.2%). All 45 patients completed conversion radiotherapy of 44.94/37.8 Gy, and 31 patients were evaluated as having a resectable tumor (Fig. 2). Among these patients, 21 cases underwent radical esophagectomy and four patients had an exploratory operation. Five patients were not fit to undergo surgery due to COVID-19, and one patient developed metastasis to the left calf muscle before surgery. Among 14 patients regarded as having unresectable disease after cCRT, 11 patients completed a definitive radiation dose of 59.92/50.4 Gy, and three patients stopped treatment at a radiation dose of 44.94/37.8 Gy due to grade 3 radiation esophagitis (two, 4.4%) and grade 1 fever (one, 2.2%). Toxicity-related deaths were not observed.

### Surgery and postoperative complications

After cCRT, 21 (46.7%) patients received curative surgery and four patients experienced exploratory surgery with a prevalence of R0 resection of 84.0% (21/25) and pCR of 47.6% (10/21). Twenty patients had a minimally invasive approach. Postoperative complications graded by the Clavien–Dindo classification are listed in Table 3. The overall prevalence of complications was 61.9% (13/21). The most common complications were pleural effusion (38.1%), pulmonary infection (33.3%), pericardial effusion (33.3%), and atelectasis (28.6%). Complications of grade ≥ IIIb were not observed. The mean duration of hospital stay was 14 days. No patients died within 30 days after surgery. The median time from the start of radiotherapy to surgery was 12.1 (IQR, 11.0–14.8) weeks.

**Table 3:** The postoperative complications and the Clavien–Dindo (CD) classification in 21 curative resection patients

Postoperative Complications	Number of patients (%) (*N* = 21)
*All complications*	34
Hemorrhage	0 (0.0)
Pulmonary infection	7 (33.3)
Pneumothorax	1 (4.8)
Atelectasis	6 (28.6)
Pleural effusion	8 (38.1)
Respiratory failure	0 (0.0)
Empyema	0 (0.0)
Arrhythmia	0 (0.0)
Heart failure	0 (0.0)
Pericardial effusion	7 (33.3)
Injury of recurrent nerve	2 (9.5)
Anastomotic leakage	1 (4.8)
Anastomotic stenosis	0 (0.0)
Incision infection	1 (4.8)
Chylothorax	0 (0.0)
*Clavien-Dindo grade*	
I	24
II	7
IIIa	3
IIIb–V	0

### Relapse

**Fig. 3:**
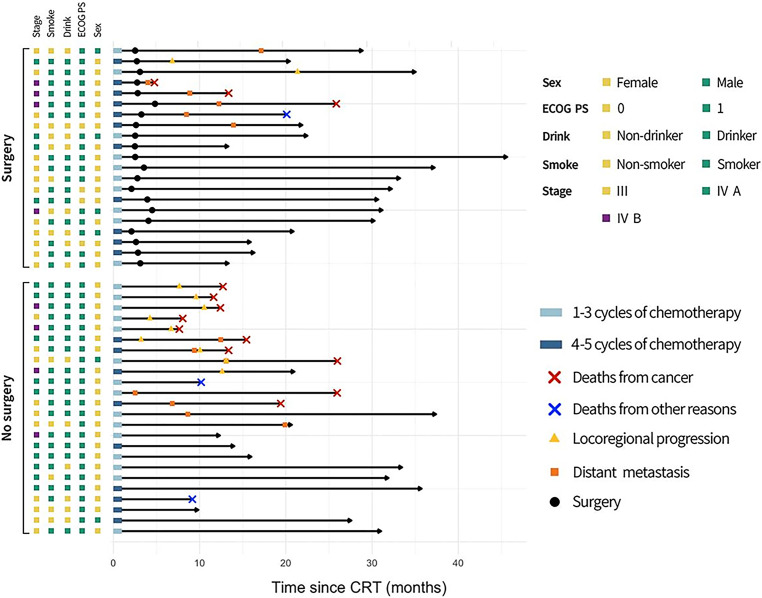
Clinical characteristics and timelines of enrolled patients. *Left cubes* represent the clinical features (sex, ECOG PS, alcohol consumption, tobacco smoking, and cTNM stage) of 45 patients. The *right timeline* records the time points of surgery (21 patients), locoregional recurrence (11 patients), distant metastasis (13 patients), and deaths (16 patients) of all patients since the start of conversion chemoradiotherapy. Abbreviations: *ECOG* *PS* Eastern Cooperative Oncology Group performance status, *CRT* chemoradiotherapy

The median duration of follow-up was 30.3 (IQR, 20.5–33.2) months. The landmark time points of disease progression and treatment are illustrated in Fig. 3. Of 45 patients, 11 patients experienced locoregional failure and 13 had distant metastasis. Thirteen patients died due to disease progression, two from heart disease, and one from an unknown disease.

**Fig. 4:**
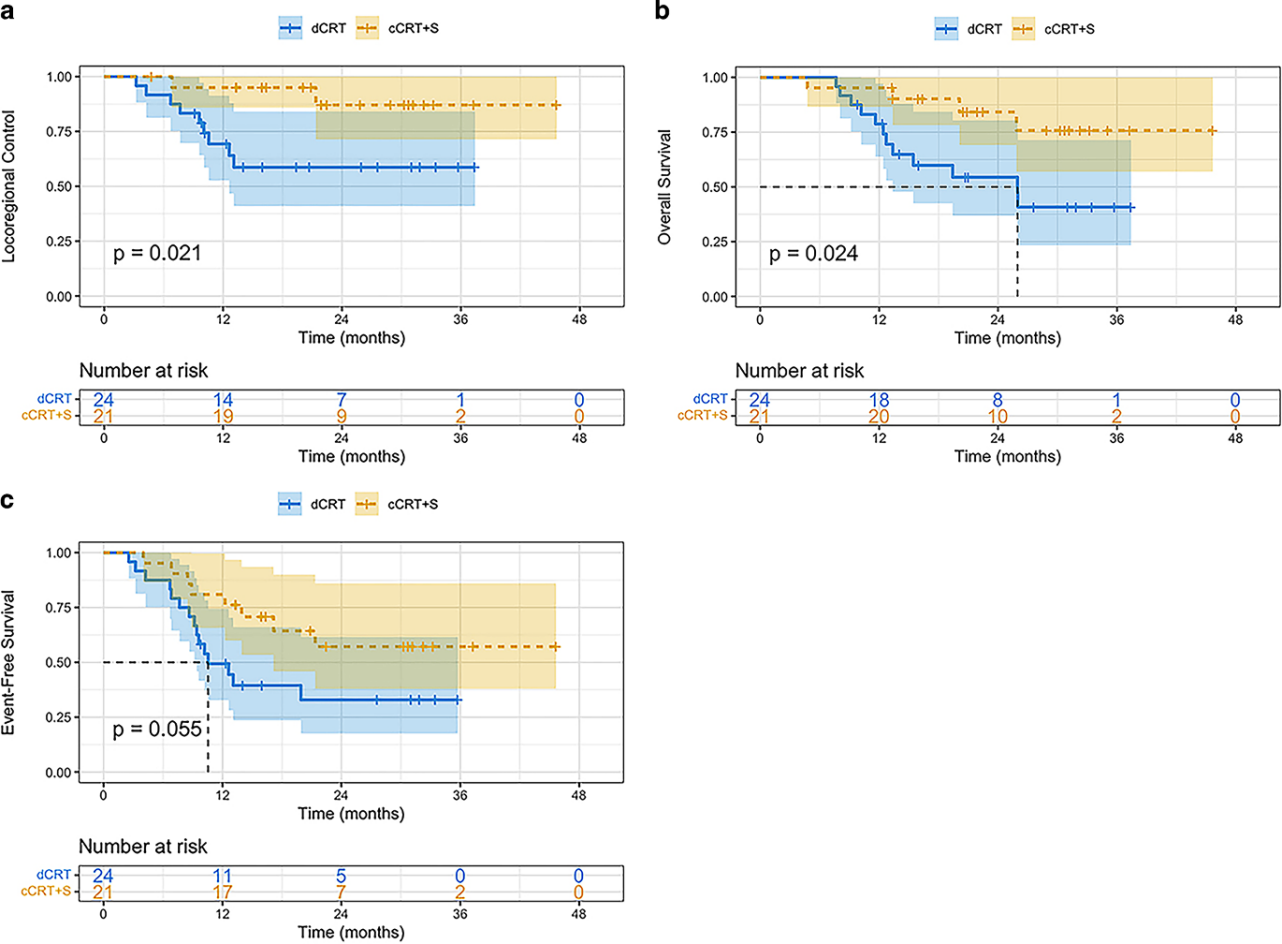
Locoregional control (**a**), overall survival (**b**), and event-free survival (**c**) of the cCRT+S group and dCRT group

The 1‑ and 2‑year LRC prevalence of this clinical trial reached 81.5 and 72.4%, respectively (Supplemental Figure S1A). The LRC prevalence at 1 and 2 years was superior in patients who had cCRT plus surgery (cCRT+S) than those who had dCRT (95.0 and 87.1% *vs*. 69.3 and 58.7%; hazard ratio (HR) = 5.14; 95%CI = 1.10–23.94; *P* = 0.021) (Fig. 4a). DMFS at 1 and 2 years was 73.2 and 52.7%, respectively (Supplemental Figure S1D).

### Survival

For the entire study cohort, the median OS was not reached (Supplemental Figure S1B). OS at 1 year and 2 years was 86.6 and 68.8%, respectively. Median OS in the cCRT+S group was not reached. Median OS in the dCRT group was 26.0 (95%CI = 13.6–38.3) months. OS at 1 year and 2 years was significantly longer in the cCRT+S group compared with that in the dCRT group (95.2 and 84.2% *vs*. 78.8 and 54.4%; HR = 3.41; 95%CI = 1.10–10.61; *P* = 0.024) (Fig. 4b).

Median EFS for all patients was 19.9 (95%CI = 9.6–30.2) months. EFS at 1 and 2 years was 64.3 and 44.8%, respectively (Supplemental Figure S1C). Median EFS was not reached in the cCRT+S group, but was 10.5 (95%CI = 6.1–14.9) months in the dCRT group (HR = 2.28; 95%CI = 0.96–5.40; *P* = 0.055) (Fig. 4c).

## Discussion

This phase I/II clinical trial determined the safety of a weekly nab-paclitaxel- and cisplatin-based regimen in cCRT in patients with locally advanced borderline-resectable or unresectable ESCC. Our study provides the first evidence that a regimen based on nab-paclitaxel and cisplatin is tolerable in cCRT for ESCC.

The newly created nab-paclitaxel has many advantages over sb-paclitaxel, such as fewer allergic reactions, no requirement for pretreatment, and a higher concentration in tumor tissue [13, 14]. With respect to first-line chemotherapy in advanced ESCC, a retrospective study demonstrated that nab-paclitaxel plus cisplatin achieved a higher objective response rate and disease control compared with sb-paclitaxel plus cisplatin [8]. In China, a regimen based on nab-paclitaxel plus platinum is used widely in ESCC as perioperative chemotherapy/radiotherapy or first-line chemotherapy. However, due to a lack of evidence-based data, a nab-paclitaxel-based chemotherapy regimen is not recommended for preoperative CRT or dCRT for esophageal cancer in CSCO guidelines.

According to the outcomes of RTOG 8501 and CROSS studies, PF and paclitaxel and carboplatin (TC) are the standard chemotherapy regimens recommended by NCCN guidelines for nCRT or dCRT of ESCC [15, 16]. In addition to PF and TC, the paclitaxel and cisplatin (TP) regimen is also standard treatment for concurrent CRT in locally advanced ESCC or first-line treatment against advanced ESCC in CSCO guidelines. Chen et al. demonstrated that the paclitaxel plus fluorouracil (TF) regimen led to similar OS with PF-based CRT against locally advanced ESCC [17]. They also showed no difference in OS among TP-, TC-, and TF-based dCRT in locally advanced ESCC, with little difference in toxicities [18]. The JCOG 1109 study demonstrated that docetaxel, cisplatin and fluorouracil (DCF) significantly improved OS and PFS compared to the standard PF regimen and PF based CRT in neoadjuvant therapy [19]. Additionally, the DCF regimen showed a superior pCR. However, the incidence of adverse effects, particularly grade 3 or higher febrile neutropenia, was significantly higher in the DCF group (16%) compared to the other two groups. Locoregional recurrence was more common in the DCF group than in the PF-based nCRT group. Another randomized trial based on paclitaxel and cisplatin showed that the pCR were similar between nCRT group (35. 7%) and neoadjuvant chemotherapy (nCT) group (3.8%); however, the nCRT group had significantly fewer deaths due to tumor progression or recurrence compared to the nCT group (6.8% vs 14.4%, *p* = 0.046) [20]. Both nCT and nCRT have their respective advantages in the treatment of esophageal cancer; incorporating radiotherapy could improve locoregional control of the disease. Nowadays, paclitaxel combined with platinum-based regimens is used widely in concurrent CRT or systemic treatment of advanced esophageal cancer in China.

In the phase II, forty-four (97.8%) patients had any grade of side-effects during cCRT, but only 11 (24.4%) patients experienced toxicities of grade ≥ 3. Radiation esophagitis was the most common toxicity of grade 3–4 (seven, 15.6%), which was comparable with other TP-based CRT studies of locally advanced ESCC (14.0–17.6%). [4, 9] Radiation pneumonia was not observed during concurrent CRT. With respect to postoperative complications, the overall prevalence in our clinical trial (61.9%) was similar to that in the nCRT group of the NEOCRTEC 5010 study (57.8%). [21] Also, the prevalence of pulmonary infection (seven, 33.3%) was similar to that observed in other studies of conversion surgery of ESCC (15.0–37.0%). [4, 22, 23] Therefore, we believe that nab-paclitaxel plus cisplatin are tolerable in terms of side-effects during radiotherapy and postoperative complications.

Locoregional recurrence after dCRT for patients with unresectable ESCC is the primary failure pattern in ~50% of patients, 83.3% of which occurs within 1 year and which carries a poor prognosis [24, 25]. Liao et al. compared dCRT and concurrent CRT plus surgery in stage-II and -III esophageal cancer [26]. They demonstrated that esophagectomy could reduce locoregional recurrence and thus lengthen OS. We obtained a 1-year LRC prevalence of 81.5% and achieved 1, 2, and 3‑year OS of 86.6%, 68.8 and 57.3%, respectively, slightly better than the results of other studies of cCRT followed by surgery (3-year OS of 29.0–31.0%). [4, 27] However, due to the insufficient sample size, firm conclusions could not be drawn. Studies have shown that if patients with unresectable tumors achieved R0 resection after conversion treatment, then the survival of these patients would be lengthened significantly [4–6, 22]. Different modes of conversion therapy for patients with borderline-resectable esophageal cancer are being investigated intensely. In particular, a combination of immunotherapy, radiotherapy, and chemotherapy have made conversion therapy more diverse. Our ongoing NEXUS study reported preliminary results in 2022 ESMO-IO, indicating that conversion therapy of concurrent chemotherapy followed by immunochemotherapy achieved a prevalence of 100% for R0 resection, 61.5% for pCR, and 76.9% for the major pathologic response.

Our clinical trial had four main limitations. First, enrollment was slow and closed prematurely due to the COVID-19 pandemic. Second, we observed improvement in locoregional control and survival, but could not confirm the efficacy of the regimen because of the small study cohort. Third, this clinical trial was conducted without combination with immunotherapy, but multiple studies have demonstrated the efficacy of immunotherapy combined with chemotherapy/radiotherapy against ESCC [28, 29]. How to combine radiotherapy, chemotherapy, and immunotherapy to improve the prognosis of patients with locally advanced borderline-resectable or unresectable ESCC merits further exploration.

## Conclusions

In our study, we demonstrated the safety and feasibility of administering nab-paclitaxel plus cisplatin-based cCRT in patients with locally advanced borderline-resectable or unresectable ESCC. Our findings indicate that this regimen does not increase surgical complications. Furthermore, our trial suggest that patients who were able to undergo subsequent surgical resection following cCRT exhibited a tendency towards improved overall survival outcomes.

## Supplementary Information

Supplemental Figure S1

Supplemental Table S1. Dose-limiting toxicities in the phase I study (N = 10)
