# Supplementary material for: Conversion chemoradiotherapy combined with nab-paclitaxel plus cisplatin in patients with locally advanced borderline-resectable or unresectable esophageal squamous cell carcinoma: a phase i/ii prospective cohort study
Source: Strahlenther Onkol. 2024 Aug 12;200(12):1038–46. doi: 10.1007/s00066-024-02286-8 (PMC11588946; doi:10.1007/s00066-024-02286-8)

**Supplemental Figure S1A**

**
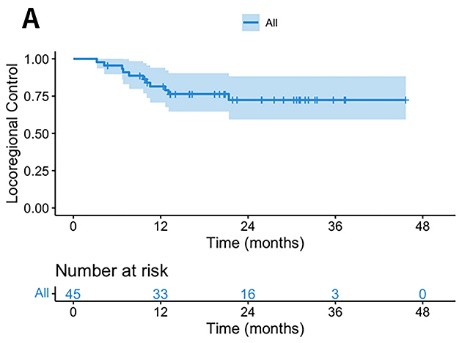
**

**Supplemental Figure S1B**


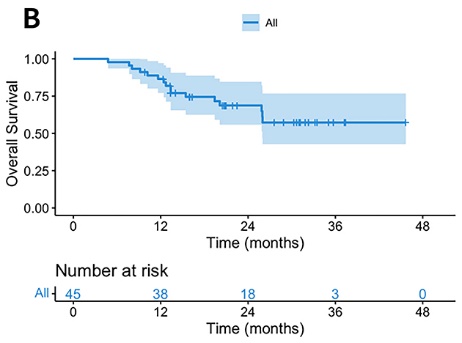


**Supplemental Figure S1C**


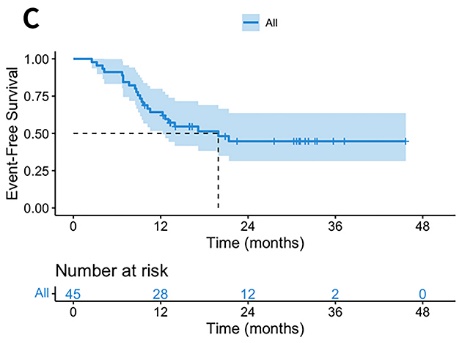


**Supplemental Figure S1D**


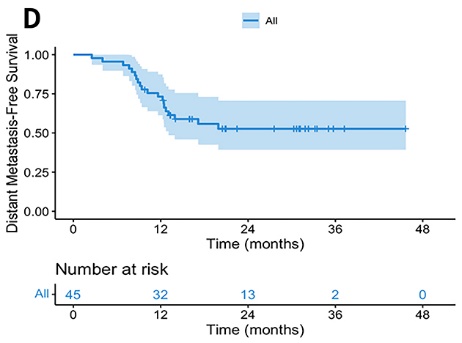

Supplement: Supplementary file 1 — Supplemental Figure S1 [file 66_2024_2286_MOESM1_ESM.docx]
