# Supplementary material for: Conversion chemoradiotherapy combined with nab-paclitaxel plus cisplatin in patients with locally advanced borderline-resectable or unresectable esophageal squamous cell carcinoma: a phase i/ii prospective cohort study
Source: Strahlenther Onkol. 2024 Aug 12;200(12):1038–46. doi: 10.1007/s00066-024-02286-8 (PMC11588946; doi:10.1007/s00066-024-02286-8)
Supplement: Supplementary file 2 — Supplemental Table S1. Dose-limiting toxicities in the phase I study (N = 10) [file 66_2024_2286_MOESM2_ESM.docx]

Supplemental Table S1. Dose-limiting toxicities in the phase Ⅰ study (N=10).

| Adverse events | Any Grade | Grade 3-4 | DLT |
| --- | --- | --- | --- |
| Fatigue | 0 | 0 | 0 |
| Nausea | 2 | 0 | 0 |
| Anorexia | 1 | 0 | 0 |
| Diarrhea | 1 | 0 | 0 |
| Radiation esophagitis | 10 | 1 | 1 |
| Radiation pneumonitis | 0 | 0 | 0 |
| Radiation dermatitis | 4 | 0 | 0 |
| Anemia | 6 | 0 | 0 |
| Neutropenia | 4 | 1 | 1 |
| Thrombocytopenia | 0 | 0 | 0 |
| Leukopenia | 10 | 1 | 1 |
| Increase in ALT/AST | 0 | 0 | 0 |

Abbreviation: DLT, dose-limiting toxicity.
